# A meta-analysis of two randomised trials of early chemotherapy in asymptomatic metastatic colorectal cancer

**DOI:** 10.1038/sj.bjc.6602841

**Published:** 2005-11-01

**Authors:** S P Ackland, M Jones, D Tu, J Simes, J Yuen, A-M Sargeant, H Dhillon, R M Goldberg, E Abdi, L Shepherd, M J Moore

**Affiliations:** 1 Australasian Gastro-Intestinal Trials Group and NSW Clinical Oncology Group, Locked Bag 77, Camperdown, NSW 1450, Australia; 2 Newcastle Mater Misericordiae Hospital, Locked Bag 7, Hunter Region Mail Centre, NSW 2310, Australia; 3 National Health and Medical Research Council, Locked Bag 77, Camperdown, NSW 1450, Australia; 4 National Cancer Institute of Canada Clinical Trials Group, 10 Alcorn Avenue, Suite 200, Toronto, Ontario, Canada M4V 381; 5 Princess Margaret Hospital, 610 University Avenue, Toronto, Canada MSG 2M9; 6 North Central Cancer Treatment Group, Operations Office, 200 First Street SW, Rochester, MN 55905, USA; 7 Department of Medical Oncology, Northern Rivers Area Health Services, Tweed Hospital, Powell Street, Tweed Heads, NSW 2485, Australia

**Keywords:** colorectal cancer, fluorouracil, quality of life

## Abstract

This report constitutes a prospectively planned meta-analysis combining two almost identical trials undertaken in Australasia and Canada to study the effect of starting chemotherapy immediately in asymptomatic patients with metastatic colorectal cancer. Patients (*n*=168) were randomised to receive either immediate or delayed treatment (at onset of predefined symptoms). Australasian patients received either weekly 5-fluorouracil and leucovorin (500 and 20 mg m^−2^, respectively) (*n*=59) or the daily × 5 Mayo Clinic schedule (425 and 20 mg m^−2^, respectively) (*n*=42). Canadian patients were treated with the Mayo schedule (*n*=67). Otherwise, the two studies were almost identical in design and each used the European Organisation for the Research and Treatment of Cancer (EORTC) QLQ-C30 instrument for measuring quality of life (QoL). Treatment was continued until 6 months had elapsed or disease progression occurred. Low accrual led to trial suspension before the predetermined sample size for either study was reached. Median survival was not significantly better with immediate treatment (median 13.0 *vs* 11.0 months; hazard ratio, 1.15; 95% confidence interval (CI) 0.79–1.72; *P*=0.49). There was no statistically significant difference in progression-free survival (time from randomisation until first evidence of progression after chemotherapy, 10.2 *vs* 10.8 months; hazard ratio, 1.08; 95% CI 0.71–1.64; *P*=0.73). There was no difference in overall QoL or its individual domains between the two treatment strategies at baseline or at any subsequent time point. Early treatment of asymptomatic patients with metastatic colorectal cancer did not provide a survival benefit or improved QoL compared to withholding treatment until symptoms occurred.

Colorectal cancer (CRC) is the most common malignancy in Western countries. Primary disease, detected early, is potentially curable surgically. However, about 20% of patients have metastatic disease at initial presentation, and about 50% of all stage A–C disease ultimately recur despite surgery and adjuvant therapy. Most of these patients have incurable metastases. Until recently, the most active cytotoxic agent against metastatic CRC was 5-fluorouracil (5FU) potentiated by leucovorin (LV, folinic acid) (Rustum, 1989). A number of clinical trials and three meta-analyses have shown improved response rates with 5FU–LV compared with 5FU alone (Piedbois *et al*, 1992; Jonker *et al*, 2000; Thirion *et al*, 2004). In addition, quality of life (QoL) can be improved as a consequence of tumour response or stabilisation by chemotherapy (Scheithauer *et al*, 1993; Glimelius *et al*, 1995). One of these meta-analyses suggested a greater treatment effect on survival advantage for poor performance status (Eastern Cooperative Oncology Group (ECOG) 2+) patients, with a treatment effect on good performance status patients that was similar (Thirion *et al*, 2004).

In asymptomatic patients, chemotherapy may be given immediately on diagnosis of incurable metastatic disease or may be withheld until symptoms develop. The appropriate timing of the use of ‘expectant’ chemotherapy has been the subject of debate, especially when the potential toxicity of treatment is taken into account. The Nordic Gastrointestinal Tumor Adjuvant Therapy Group (1992) addressed this issue in a randomised controlled trial in 183 patients with advanced but asymptomatic CRC. The results of this study suggested a survival benefit for patients receiving early treatment with methotrexate and 5FU–LV (MFL), but there were methodological issues: only 57% of patients randomised to expectant treatment received chemotherapy; chemotherapy was not standardised; outcomes were poorer than expected in the expectant treatment group; and no formal QoL analyses were published.

In a separate study from Finland, the response rate to an epirubicin, methotrexate and 5-FU combination was much greater in asymptomatic patients with advanced CRC than in symptomatic patients (40 *vs* 4%, *P*<0.001), and survival correlated with performance status, but this was a *post hoc* analysis in a nonrandomised trial (Pyrhonen and Kouri, 1992).

Inconclusive results from these two studies and a range of opinions among medical oncologists who treat CRC led us to evaluate whether asymptomatic patients should receive chemotherapy immediately or could defer therapy without compromising outcomes. Two almost identical prospective, randomised controlled trials were undertaken by the Australasian Gastrointestinal Trials Group (AGITG) and the NSW Clinical Oncology Group, and the National Cancer Institute of Canada (NCIC) Clinical Trials Group. The primary aim of both studies was to evaluate the effect of the early use of 5FU–LV on survival of asymptomatic patients with advanced CRC. Quality of life was a secondary outcome. We report here a preplanned, prospective meta-analysis of survival and QoL of the two studies.

## PATIENTS AND METHODS

### Patient population

Patients were recruited from 17 hospitals in Australasia (Australia and New Zealand) and 17 hospitals in Canada between May 1994 and October 1999. Patients in both studies were required to have histologically proven CRC with local recurrence or metastases not amenable to curative treatment. Patients were required to have no symptoms related to advanced disease, specified as: no pain requiring regular narcotic analgesics; no weight loss over 5 kg (unless related to surgery or other illness); no persistent nausea requiring medication; ECOG performance status of 0 or 1 (Canada: Karnofsky performance status >90%); no obstructive bowel symptoms; no persistent fever related to metastatic cancer; and no other symptom which in the opinion of the clinician was due to progressive metastatic cancer. Other eligibility criteria included: no prior chemotherapy for metastatic disease (patients might have received 5FU-based adjuvant treatment more than 6 months before the development of metastatic disease); evaluable or measurable disease according to the criteria of Miller (Miller *et al*, 1981); creatinine <150 *μ*m l^−1^ or creatinine clearance >1 ml s^−1^; bilirubin <25 *μ*m l^−1^, aspartate aminotransferase (AST) <2 × upper limit of normal (ULN), alanine transferase (ALT) <3 × ULN; granulocytes >1.5 × 10^9^ l^−1^, platelets >100 × 10^9^ l^−1^; no central nervous system metastases; no history of prior invasive malignant disease; and no uncontrolled medical condition that would be aggravated by treatment. Women of childbearing potential had to be not pregnant and agree to adequate contraception for the duration of the study. The study was approved by local institutional ethics committees, and written informed consent was obtained.

Pretreatment evaluation included patient history, physical examination, clinical tumour measurement, full blood cell count, electrolytes, urea, creatinine, liver function tests (bilirubin, AST, ALT), albumin, calcium and glucose, chest X-ray, electrocardiogram and radiological investigations as indicated to document evaluable or measurable disease. Serum lactate dehydrogenase measurement was mandated in Australasian patients only.

### Randomisation and treatment

Patients were stratified according to treatment centre and prior adjuvant chemotherapy (yes or no), then randomised to immediate treatment (to begin within 2 weeks) or treatment delayed until the development of symptoms.

The treatment schedule differed in the Australasian and Canadian studies. In Canada, the study allowed only the Mayo clinic schedule: (5FU 425 mg m^−2^ intravenous (i.v.) bolus on days 1–5, LV 20 mg m^−2^ i.v. bolus on days 1–5, repeated every 28 days). In Australasia, clinicians chose between the Mayo clinic schedule or weekly 5FU–LV (5FU 500 mg m^−2^ i.v. bolus and LV 20 mg m^−2^ i.v. bolus), but nominated the schedule before randomisation (Buroker *et al*, 1994; Kerr *et al*, 2000). Every attempt was made to treat all patients for at least 2 months.

For patients allocated to delayed treatment, chemotherapy was started when defined criteria were met. These criteria included a decline in performance status to ECOG ⩾2 or Karnofsky <90%, weight loss more than 4.0 kg from the time of study entry, persistent nausea requiring medication, pain requiring regular narcotic analgesics, elevation of bilirubin to >25 *μ*m l^−1^ due to metastatic disease, elevation of AST or ALT to >3 × ULN, other symptoms due to progressive cancer, development of clinically significant third-space fluid collections or if it was felt that further delays in instituting chemotherapy would be unwise.

Dose modifications were undertaken according to the toxicity in the previous cycle. Patients with no toxicity at all could have the 5FU dose increased by 50 mg m^−2^. Grade ⩽2 nonhaematological toxicity or nadir neutrophil counts ⩾0.5 × 10^9^ l^−1^ or platelets ⩾50 × 10^9^ l^−1^ did not require dose modification. Grade 3 nonhaematological toxicity or nadir neutrophil counts <0.5 × 10^9^ or platelets <50 × 10^9^ l^−1^ warranted 5FU dose reduction by 50 mg m^−2^. Any grade 4 toxicity warranted reduction by 100 mg m^−2^. Patients whose measures on laboratory parameters had not returned to pretreatment levels at the time of the next cycle or week of therapy had treatment withheld weekly for a maximum of 2 weeks to allow recovery.

### Evaluations of patients

All patients were reviewed monthly throughout the study period with history and physical examination, performance status, full blood cell count and serum biochemistry. Assessments of QoL were completed every 2 months and tumour imaging every 3 months. Patients with responding disease (assessed by standard criteria; Miller *et al*, 1981) continued on treatment. Treatment was discontinued if there was evidence of disease progression, intolerable toxicity despite dose reductions, completion of six cycles of chemotherapy with best response being only stable disease, or at the patient's request.

In both trials, QoL assessment was undertaken every 2 months to provide a direct comparison of QoL with and without chemotherapy. Both studies used the European Organisation for the Research and Treatment of Cancer (EORTC) QLQ-C30, which is a well-validated, comprehensive, core questionnaire consisting of 30 items that assess function, symptoms and overall QoL (Aaronson *et al*, 1991). The Canadian questionnaire had three more questions than QLQ-C30. Identical questions were combined for the analysis.

Additional instruments used in Australasia included the linear analogue self-assessment (LASA) scales of Priestman and Baum (1976), the GLQ-8 scales of Coates *et al* (1983) and the QL index of Spitzer *et al* (1981).

### Statistical analysis

Continuous variables were presented as medians with interquartile ranges. Australasian and Canadian studies were combined in a prospective meta-analysis for the primary outcome of survival and also time to ultimate disease progression (time from randomisation until first evidence of progression after chemotherapy). Risk factors considered in univariate and multivariate analysis of predictors of survival and time to ultimate progression included age, sex, performance status, weight, stage at diagnosis and disease-free interval. Outcomes were analysed with the Cox proportional-hazards regression model (with stratification by trial for combined analysis), with hazard ratio and 95% confidence intervals (CIs) reported. Relevant variables were adjusted for in a multivariate model. Survival curves were presented as Kaplan–Meier plots. All analyses used SPIDA and SAS (versions 7 and 8) software, classified data by intention to treat, and were two-sided with a 5% level of significance.

Combined data for baseline-adjusted EORTC QLQ-C30 scores for overall QoL and individual domains of QoL for treatment groups were compared. Quality of life scores are rarely normally distributed, and the data distributions in this study were skewed; presenting means and standard deviations was not appropriate. Therefore, the data were dichotomised into ‘good’ and ‘poor’ categories, with proportions and 95% CIs presented graphically for the ‘good’ QoL scores by treatment group and time. Alternatively, medians and interquartile ranges could have been presented; however, the scales were small and presenting medians would not show subtle differences.

The primary objective of each trial was to detect differences in overall survival. The intended combined sample size of 400 patients had 83% power to detect a 15% absolute improvement in 1-year survival from 40 to 55% at a 5% significance level. As both trials collected common QoL data using the QLQ-C30 questionnaire, there was good power (>90%) to detect small differences in QoL from the combined studies. With the final sample size of 168 patients (median follow-up of 57 months), the combined analysis would have an 80% power to detect a 20% improvement in 1-year survival (from 45 to 65%).

## RESULTS

### Patient characteristics and treatment

**Figure 1:**
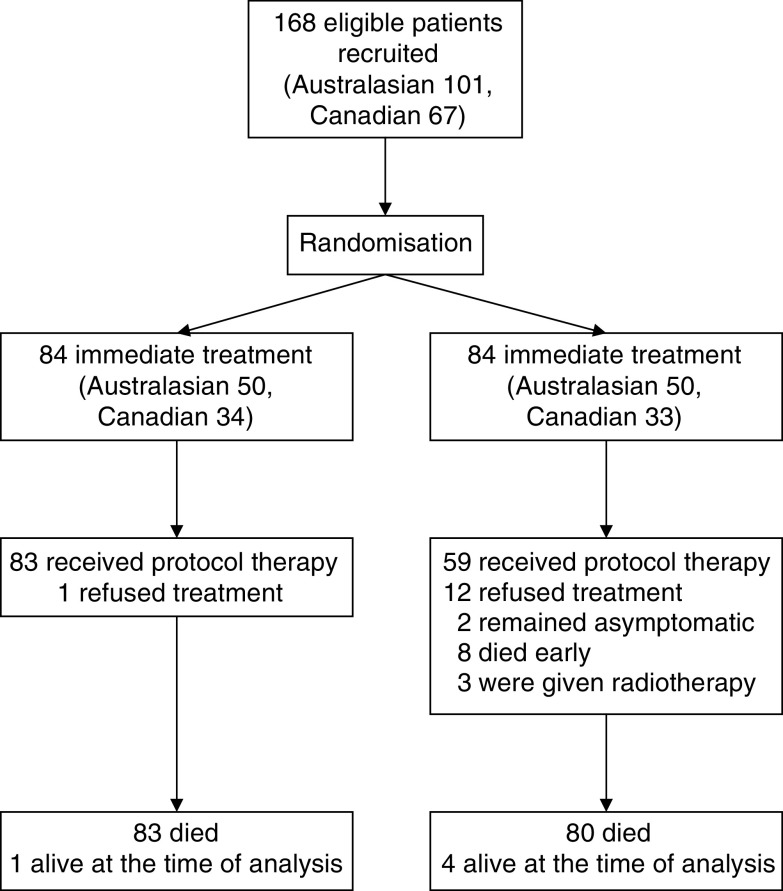
Progress of patients in the Australasian and Canadian colorectal cancer trials.

Owing to slow accrual, recruitment was suspended before the predetermined sample size for each study was reached. However, the prospective meta-analysis was still conducted as intended. A total of 168 patients were recruited (101 Australasians, 67 Canadians) (Figure 1). Patient demographic characteristics and factors of potential prognostic importance were generally well balanced between the immediate- and delayed-treatment arms (Table 1). The median age was similar across immediate and delayed groups for both studies, although there were more patients aged over 70 years in the immediate-treatment groups. All Canadian patients had ECOG performance status 0, whereas 21% of Australasian patients had ECOG performance status 1 on the basis of comorbidity. All Australasian patients had measurable disease, whereas 26% of Canadian patients had only evaluable, nonmeasurable disease. The most common sites of metastases were liver and lung.

Only one patient randomised to the immediate-treatment group did not receive protocol therapy, deciding to defer treatment until the disease had progressed. In the delayed-treatment group, 59 (70%) ultimately received protocol (5FU–LV) therapy. Of the remaining 25 patients in the delayed-treatment group, 12 declined chemotherapy at the decision point and were subsequently managed by supportive care (seven), radiotherapy (one), immediate chemotherapy (one), other chemotherapy in a research study (one), and care unknown (two). Two patients had not developed symptoms by 55 and 57 months after randomisation, respectively. Eight patients died before starting therapy 2.2–53 months after randomisation; of these, seven died of progressive disease and one died of postoperative complications after presenting with acute bowel obstruction related to peritoneal metastases. Three patients were treated with radiation therapy alone.

No patients received second-line systemic treatments, since neither irinotecan nor oxaliplatin were available during the study period.

All Canadian patients received the Mayo clinic schedule of four-weekly 5FU–LV (Table 2). In the Australasian cohort, 22 of 49 patients in the immediate-treatment group and 12 of 33 treated patients in the delayed-treatment group received weekly 5FU–LV. In the delayed-treatment group, the median time from randomisation to treatment initiation was 6 months (range 1–34) in the Australasian cohort and 4 months (1–28) in the Canadian cohort. Reasons for starting treatment in the delayed-treatment group were onset of symptoms (pain (23%), declining performance status (29%), weight loss (20%), nausea (6%), elevated bilirubin (4%), elevated transaminases (6%)), and significant disease progression without protocol-specified increased symptoms (13%).

### Time to disease progression

The time to disease progression from randomisation to after chemotherapy was similar in the immediate- and delayed-treatment groups for both studies (Table 3). Pooling the results of the two studies by meta-analysis (test for heterogeneity, *P*=0.047) did not disclose a difference in time to disease progression between the immediate- and delayed-treatment groups. Disease progression occurred at a median of 10.2 months after randomisation in patients receiving immediate treatment, and 10.8 months in the delayed treatment group (hazard ratio, 1.01; 95% CI 0.72–1.41). After adjustment for age, sex, weight, ECOG performance status, prior adjuvant chemotherapy, and baseline QoL, the hazard ratio was similar.

### Survival

In September 2004, the median follow-up time was 55 months, and 163 patients had died (83 in the immediate-treatment group and 80 in the delayed-treatment group). In all, 89% of patients died from tumour progression, 3% from treatment-related toxicity, and 8% from other unrelated conditions.

**Figure 2:**
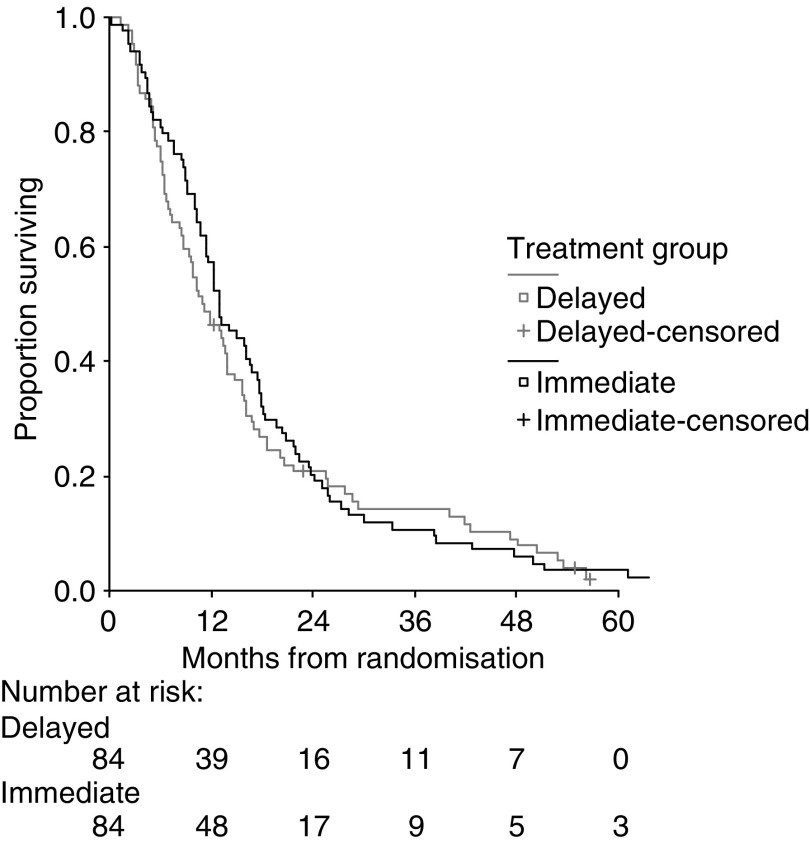
Survival of patients after randomisation to immediate or delayed chemotherapy treatment for colorectal cancer (*P*=0.6): pooled data from the two trials.

Survival after study entry was similar in the immediate- and delayed-treatment groups for both the Australasian and Canadian studies (Table 3). Pooling the results of the two studies by meta-analysis (test of heterogeneity, *P*=0.5) did not show a difference in survival between the groups. The 1-year survival was 57% for immediate treatment and 46% for delayed treatment. Median survival was 13.0 months in patients receiving immediate treatment and 11.0 months in the delayed-treatment group (hazard ratio, 1.08; 95% CI 0.79–1.47) (Figure 2). Adjustment for risk factors did not significantly change the hazard ratio (hazard ratio, 1.15; 95% CI 0.77–1.72).

Multivariate analysis was conducted with the Cox proportional-hazards model. Age, sex, liver metastases, ECOG performance status, baseline QoL scores, weight, disease-free interval >12 months, stage at first presentation, and prior adjuvant chemotherapy did not statistically significantly influence time to disease progression or survival for immediate *vs* delayed treatment.

### Quality of life

**Figure 3:**
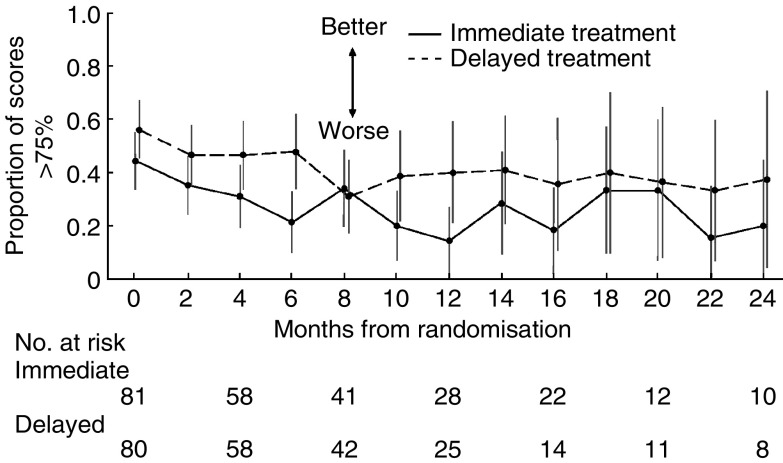
Proportion of patients rating their global health status score higher than 75% (on EORTC QLQ-C30) at each time point after randomisation to immediate or delayed chemotherapy treatment for colorectal cancer. A high score indicates better quality of life. The vertical lines show 95% CIs. The data points have been offset for clarity. The number at risk is the number of patients who completed questionnaires.

Patients completed QoL assessments every 2 months from randomisation until death. The EORTC QLQ-C30 was common to both the Australasian and Canadian studies. Form completion rates were high at baseline and at each subsequent time point. Quality of life was similar for patients in both treatment arms at baseline. All 168 patients completed questionnaires at baseline, with over 95% of all questions answered. At 12 months, 85% of living, immediately treated patients and 74% of living patients allocated to delayed treatment completed questionnaires. For global health status, a score higher than 75% was considered to indicate ‘good’ QoL. Figure 3 shows the proportion of ‘good’ scores over time by treatment group; a proportion of 0.5 indicates that half the patients had QoL scores higher than 75%. Global health status in the immediate treatment group was nonsignificantly lower at all time points, except at 8 months, when QoL was the same. There was no statistically significant difference in overall QoL or any component domain between the two treatment strategies at baseline or at any subsequent time point.

The use of the LASA scales of Priestman and Baum (1976), the GLQ-8 scales of Coates *et al* (1983) and the QL index of Spitzer *et al* (1981) showed no differences from the results of the EORTC scales. Physical well-being (LASA) was rated high at baseline (median 1 cm on a 10 cm scale) with nonsignificant deterioration over time (median 2.2 cm at 8 months) with no difference between immediate- and delayed-treatment groups (data not shown). Scales for pain, appetite, thought of feeling sick, nausea and vomiting, and overall QoL showed no differences between treatment groups or over time. The proportion of patients with a QL index score of 5 (optimal QoL) fell from 60% at baseline to 40% by 14 months, but with no significant difference between the treatment strategies or over time (data not shown). The immediate- and delayed-treatment groups had no significant difference at any time point in most of the individual domains of QoL in the EORTC and other instruments (ECOG performance status, dyspnoea, loss of appetite, constipation, diarrhoea, pain, fatigue, nausea and vomiting, and physical, role functioning, emotional, and cognitive functioning). The two exceptions were insomnia and social functioning, in which those patients in the immediate-treatment group showed a nonsignificant trend for poorer QoL over time compared with those in the delayed-treatment group.

### Adverse effects

Treatment toxicities were as expected for these regimens of 5FU–LV (Table 4), with no statistically significant differences in incidence or severity of worst side effects between immediate- and delayed-treatment groups. Neutropenia occurred in 14% of patients and infection was uncommon (6% of patients overall), with no significant difference between patient groups. Grade 3 or 4 stomatitis occurred in 13% of patients, with no difference between the two arms. Grade 3 or 4 diarrhoea occurred in 20% of patients overall and was more common in immediately treated patients. For Australasian patients, a comparison of toxicities for the two different schedules indicated more grade 3 or 4 diarrhoea with weekly treatment (24 *vs* 13%), but less grade 3 or 4 neutropenia (3 *vs* 9%) and stomatitis (2 *vs* 8%).

## DISCUSSION

In this analysis, no survival benefit could be demonstrated from the use of immediate 5FU–LV treatment of asymptomatic patients with metastatic CRC, compared to a policy of watchful waiting and institution of 5FU–LV when symptoms occurred. This result occurred even though 25 (30%) patients allocated to delayed treatment did not receive any chemotherapy. Imbalances in characteristics between groups were minor and had no significant effect. Patients on immediate treatment tended to be older, but adjustment for risk factors including age, performance status, prior adjuvant chemotherapy, and baseline QoL did not change the results of the analysis.

Quality of life was not enhanced or adversely affected by immediate treatment. The data show no significant difference in overall QoL or any individual domain of QoL between the two groups, at any time point. Up to the 12-month time-point completion rates of QoL questionnaires were high and almost identical between the two treatment arms, indicating reliability of interpretation. However, beyond 12 months, differences in completion rates between the two study arms make interpretation of QoL data difficult, since reasons for noncompletion may vary between the two treatment strategies.

The patient population studied is typical of the cohort of patients with metastatic CRC that present without symptoms. All our patients were of good performance status with 88% of patients overall having ECOG performance status zero. In the delayed-treatment group, the median time to the development of symptoms requiring treatment was 5 months with a very wide range (1–34 months), which was similar to the Nordic trial (179 days, range 5–901 days) (Nordic Gastrointestinal Tumor Adjuvant Therapy Group, 1992). However, the result of our study is at odds with the Nordic study, which showed a longer survival for immediate treatment than for delayed treatment. In that study, patients were allocated to receive immediate treatment with 12 courses of MFL (methotrexate, LV, and 5FU), or to ‘primary expectancy’, with chemotherapy withheld until symptoms occurred. The survival for immediately treated patients in our study was similar to the Nordic study, but for delayed-treatment patients, outcome data were worse in the Nordic study; patients receiving delayed treatment had shorter symptom-free survival compared to immediate treatment (2 *vs* 10 months, *P*<0.001), poorer survival (median 9 *vs* 14 months, *P*=0.02), and lower 1-year survival (38 *vs* 55%, *P*=0.03). In our study, delayed treatment patients had a median time to start of treatment (similar to symptom-free survival) of 5 months and a median survival of 11.0 months, somewhat longer than the Nordic study. In the Nordic study, two-monthly review may have compromised the outcome for delayed patients since significant health deterioration is more likely to have occurred in the longer review interval compared to our study. Alternatively, subtle differences in characteristics of patients may account for the differences in results of the two studies.

At the time our two studies were conceived, we deliberately intended to perform a meta-analysis to improve sensitivity of both QoL and survival assessments. Therefore, each study collected essentially identical data. Unfortunately, it was not considered feasible to include the Nordic study data into this meta-analysis for a number of reasons. Firstly, this was a preplanned analysis, free of any bias as a consequence of knowledge of the individual trial results. Secondly, the Nordic trial was the study that generated the questions being asked in these two trials. Thirdly, no formal comparable QoL assessments were undertaken in the Nordic trial and the chemotherapy used was not standardised in the delayed-treatment arm. However, it is possible to undertake a simplified fixed-effects meta-analysis with the end point of 12-month survival using our two studies as prospective evidence and the three studies as total evidence, since this end point is available for all three studies. The results are presented as Table 5, and show a nonsignificant trend towards improved 12-month survival for immediate treatment (odds ratio 1.63, 95% CI 0.71–3.7, *P*=0.25).

Slow accrual was a problem in our study, preventing it from either definitively supporting or refuting the results of the Nordic study. Many patients and physicians had a preference for either immediate or delayed treatment and this limited accrual to the study, even though there was equipoise among oncologists as to which strategy was appropriate. The small sample size in this study has resulted in insufficient power to detect modest differences in survival and QoL between immediate- and delayed-treatment schedules; by chance, we could have missed a 20% difference in 1-year survival. Nonetheless, the minor trend towards better survival, lack of trend towards better progression-free survival, and lack of effect on QoL with immediate treatment suggests that if real benefits exist for early treatment they are small.

This study demonstrates the problems of compliance with a management plan involving delayed therapy in patients with terminal cancers. Only 70% (59 of 84) of patients in the delayed-treatment group actually received the protocol-defined chemotherapy, which was marginally higher than the Nordic study (57%, 51 of 90). One-quarter of our patients not receiving chemotherapy died early, or were too ill to receive treatment, and one-third chose alternative treatments. Such compliance issues would tend to bias this study in favour of immediate treatment. This issue should be considered in planning any future studies of chemotherapy in this context.

This study was begun in the early 1990s to address the fundamental question of timing of chemotherapy in patients with asymptomatic cancer. At the time the study was conducted, the thymidylate synthase inhibitors such as 5FU were the only treatments of demonstrated benefit. The introduction of additional agents has expanded the range of options for these patients and debate about the timing of the institution of chemotherapy has been overtaken by a desire to incorporate these new agents to achieve the greatest patient benefit (Simmonds, 2000). However, the newer regimens also bring more toxicity, so the question as to whether asymptomatic patients with advanced CRC should be treated remains unanswered (Simmonds, 2000). Certainly the increased toxicity of irinotecan and oxaliplatin would have greater adverse effects on QoL in asymptomatic patients, which would need to be offset by considerable survival gains to make such treatment worthwhile in this setting. Two recently reported studies throw some light on this issue. The MRC FOCUS randomised study accrued 2135 patients and compared a reference treatment of modified de Gramont schedule 5FU–LV followed by irinotecan upon primary treatment failure, with four other regimens using either second-line combination therapy or first-line combination therapy (Seymour, 2005). None of the combination regimens was superior to the reference treatment in terms of overall survival even though a higher response rate was seen. This study suggests that there is no detriment to use of 5FU–LV as initial treatment in an asymptomatic patient, reserving more toxic combination regimens for second-line management. The LIFE study compared an infusional 5FU–LV regimen alone with the same regimen plus oxaliplatin in 725 patients (Pluzanska *et al*, 2005). While the combination regimen showed better response rate (54 *vs* 30%, *P*<0.0001) and progression-free survival (7.9 *vs* 5.9 months, *P*<0.0001), there was no difference in survival (15.9 *vs* 15.2 months). Again, these data suggest that 5FU alone is acceptable as initial management in advanced colorectal cancer, particularly since QoL is likely to be more greatly affected by a combination regimen. To date, data on the effect of palliative chemotherapy on QoL in CRC are insufficient (Conroy *et al*, 2003). A large, randomised controlled trial in asymptomatic patients would be needed to establish the benefit of newer treatments on patient and cancer outcomes in this subgroup. In the meantime, the results of the study reported here suggest that immediate treatment of asymptomatic patients with 5FU–LV is not necessary to maximise QoL or survival, and that close monitoring and chemotherapy when symptoms prevail is a reasonable management strategy.

## Figures and Tables

**Table 1:** Characteristics at baseline of patients with asymptomatic colorectal cancer in two trials

	**Australasia**		**Canada**	
	**Immediate**	**Delayed**	**Immediate**	**Delayed**
**Characteristic**	***n*=50**	***n*=51**	***n*=34**	***n*=33**
Median age (years)	66.8	65.3	67.4	63.7
Age range (years)	46–80	36–77	56–80	50–78
Aged >70 years (*n*, %)	17 (34)	8 (16)	13 (38)	7 (21)
Female (*n*, %)	12 (24)	14 (27)	8 (24)	9 (27)
				
*ECOG performance status*				
0 (*n*, %)	42 (84)	38 (75)	34 (100)	33 (100)
1 (*n*, %)	8 (16)	13 (25)	0	0
				
*Karnofsky performance status*				
100 (*n*, %)	NA	NA	14 (41)	21 (64)
90 (*n*, %)	NA	NA	20 (59)	12 (36)
				
Measurable disease	50 (100)	51 (100)	24 (71)	25 (76)
*Sites of disease* a				
Peritoneal cavity (*n*, %)	NA	NA	5 (15)	4 (12)
Liver (*n*, %)	39 (83)	34 (71)	23 (68)	24 (73)
Lung (*n*, %)	10 (21)	17 (35)	12 (35)	10 (30)
Lymph nodes (*n*, %)	7 (15)	9 (19)	NA	NA
Other (*n*, %)	9 (19)	11 (23)	21 (62)	12 (36)
Weight loss (*n*, %)	7 (14)	9 (18)	NA	NA
Prior adjuvant chemotherapy (*n*, %)	14 (28)	12 (24)	9 (26)	8 (24)
Stage IV at first presentation (*n*, %)	19 (38)	12 (24)	NA	NA
Disease-free interval >12 months (*n*, %)	NA	NA	14 (41)	13 (39)
Treatment-free interval (months)	NA	NA	9–58	13–46

NA=not available.

a Patients could have more than one site of disease.

**Table 2:** Treatment received by patients with asymptomatic colorectal cancer in two trials

	**Australasia**		**Canada**	
	**Immediate**	**Delayed**	**Immediate**	**Delayed**
**Treatment details**	***n*=50**	***n*=51**	***n*=34**	***n*=33**
Protocol chemotherapy (*n*, %)	49 (98)	33 (65)	34 (100)	26 (79)
				
*Regimen*				
Weekly FU–LV (*n*, %)	22 (44)	12 (24)	NA	NA
4-weekly FU–LV (*n*, %)	27 (54)	21 (41)	34 (100)	26 (79)
Number of treatment cycles (median, interquartile range)	6 (3–8)	4.5 (1.5–7.5)	6 (3–8)	3 (2–6)
Delay to treatment start (median, range, in months)^a^	0 (0–1)	6 (1–34)	0 (0)	4 (1–28)

NA=not available; FU–LV=5-fluorouracil and leucovorin.

a Only patients who began treatment are included in this analysis.

**Table 3:** Median time to event for patients with asymptomatic colorectal cancer in two trials

	**Immediate treatment**	**Delayed treatment**		
**Outcome**	***n*=84**	***n*=84**	**Hazard ratio (95% CI)**	***P*-value**
*Time to disease progression (months)*				
Australasian trial	13.0	12.0	1.11 (0.72–1.70)	0.65
Canadian trial	7.7	8.4	0.89 (0.52–1.51)	0.65
Total	10.2	10.8	1.01 (0.72–1.41)	0.95
Adjusted^a^			1.08 (0.71–1.64)	0.73
				
*Survival (months)*				
Australasian trial	15.5	11.9	1.08 (0.73–1.62)	0.70
Canadian trial	11.9	10.2	1.07 (0.66–1.76)	0.78
Total	13.0	11.0	1.08 (0.79–1.47)	0.63
Adjusted^a^			1.15 (0.77–1.72)	0.49

CI=confidence interval.

a Adjusted for age, sex, weight, ECOG performance status, prior adjuvant chemotherapy, and baseline quality of life.

**Table 4:** Number of patients with asymptomatic colorectal cancer in two trials having grade 3 or 4 toxicity

	**Australasia**		**Canada**	
	**Immediate**	**Delayed**	**Immediate**	**Delayed**
**Type of toxicity (*n*, %)**	***n*=49^a^**	***n*=31^a^**	***n*=34^a^**	***n*=26^a^**
Nausea and vomiting	3 (6)	5 (16)	6 (18)	0 (0)
Stomatitis	1 (2)	4 (13)	9 (26)	4 (15)
Diarrhoea	10 (20)	5 (16)	9 (26)	4 (15)
Dermatitis	0 (0)	0 (0)	0 (0)	1 (4)
Infection	4 (8)	0 (0)	3 (9)	2 (8)
Other	3 (6)	2 (6)	10 (29)	12 (46)
Haemoglobin	0 (0)	1 (3)	0 (0)	2 (8)
White blood cells	0 (0)	0 (0)	3 (9)	5 (19)
Neutrophils	4 (8)	1 (3)	8 (24)	6 (23)
Platelets	0 (0)	1 (3)	0 (0)	0 (0)
Creatinine	0 (0)	0 (0)	1 (3)	0 (0)
Bilirubin	1 (2)	2 (6)	0 (0)	5 (19)
Alkaline phosphatase	1 (2)	7 (23)	0 (0)	1 (4)
Aspartate aminotransferase	0 (0)	0 (0)	0 (0)	0 (0)
Lactate dehydrogenase	7 (14)	7 (23)	NA	NA

NA=not applicable.

a Number receiving protocol therapy.

**Table 5:** Comparison of proportion surviving at 12 months

**Study**	**Immediate, *n*/*N* (%)**	**Delayed, *n*/*N* (%)**	**OR**	**95% CI**	***P*-value**
Nordic	51/93 (55)	34/90 (38)	2.04	1.13–3.7	0.03
Australasian	31/50 (62)	24/51 (47)	1.84	0.83–4.1	0.13
Canadian	17/34 (50)	15/33 (45)	1.20	0.46–3.1	0.7
All studies^a^			1.63	0.71–3.7	0.25
Australia and Canada^b^			1.40	0.47–4.1	0.5

OR=odds ratio; CI=confidence interval.

a Tests for heterogeneity at *P*=0.8.

b Tests for heterogeneity at *P*=0.7.

## Appendix A1

### CO.10 trial team

Dr Malcolm Moore, NCIC CTG Study Chair; Dr Richard M Goldberg, NCCTG Study Chair; Dr Alan K Hatfield, NCCTG Study Chair; Dr Christine Cripps, Dr Christopher de Gara, Dr Bernard Langer, Dr John Pedersen, Dr Alfred Wong, Dr Neill Iscoe, NCIC CTG Quality of Life Coordinator; Dr Lois Shepherd, NCIC CTG Physician Coordinator; Dr Dongsheng Tu; Ms Ann-Marie Sargeant.

### NCIC CTG coordinating centre staff

Dr Lois Shepherd, Physician Coordinator; Ms Ann-Marie Sargeant, Study Coordinator; Dr Dongsheng Tu, Senior Biostatistician; Mr Eric Bacon, Biostatistician; Mrs Julia Baran, Clinical Trial Assistant; Mrs Vicki Classen, Assistant.

### NCCTG staff

Ms Jeannine A Hadley, Study Coordinator; Dr Daniel Sargent, Biostatistician; Dr Heff Sloan, Quality of Life Coordinator.

### AGITG trial team

Professor Stephen Ackland (Study Chairman), Newcastle Mater Hospital; Dr David Bell, Royal North Shore Hospital; Professor Michael Findlay, Wellington Hospital; Professor John Simes, NHMRC Clinical Trials Centre, University of Sydney; A/Professor Michael Solomon, Royal Prince Alfred Hospital; Professor John Zalcberg, Peter MacCallum Cancer Institute; Mr Mark Jones, NHMRC Clinical Trials Centre; Ms Jennifer Yuen, NHMRC Clinical Trials Centre; Ms Haryana Dhillon, NHMRC Clinical Trials Centre.

### AGITG coordinating centre, NHMRC Clinical Trials Centre, staff

Ms Elaine Beller, Biostatistician, Trial Manager; A/Professor Val Gebski, Senior Biostatistician; Mr Mark Jones, Biostatistician; Ms Jennifer Yuen, Study Coordinator; Ms Yan Ping Zhang, Study Coordinator; Ms Haryana Dhillon, Trial Manager; Ms Yeon Suk Baik, Computer Programmer; Ms Sinden Medley, Assistant; Dr Martin Stockler, Clinical Epidemiologist; Professor John Simes, Biostatistician and Clinical Trialist.

### Contributing investigators

*CO.10* Dr J Skillings, QEII Centre for Clinical Research, Halifax; Dr B Leyland-Jones, McGill University, Montreal; Dr M Belanger, CHUQ- Pavillon Dieu de Quebec, Quebec; Dr J Latreille, CHUM – Hotel Dieu du Montreal, Montreal; Dr B Findlay, Hotel Dieu Health Sciences Hospital, Niagara, St Catharines; Dr J Giesbrecht, Hotel Dieu Health Sciences Hospital, Niagara, St Catharines; Dr M Samosh, Hotel Dieu Health Sciences Hospital, Niagara, St Catharines; Dr C de Gara, Hamilton Regional Cancer Centre, Hamilton; Dr A Figueredo, Hamilton Regional Cancer Centre, Hamilton; Dr LA Zibdawi, Southlake Regional Health Centre, Newmarket; Dr L Rotstein, University Health Network – Princess Margaret Hospital, Toronto; Dr L Rudinskas, Humber River Regional Hospital, Toronto; Dr JJ Wilson, Humber River Regional Hospital, Toronto; Dr R Feld, University Health Network – Princess Margaret Hospital, Toronto; Dr Malcolm Moore, University Health Network – Princess Margaret Hospital, Toronto; Dr D Walde, Algoma Regional Cancer Program, Sault Area Hospital, Sault Ste. Marie; Dr S Akhtar, Allan Blair Cancer Centre, Regina; Dr C Fitzgerald, Allan Blair Cancer Centre, Regina; Dr T Tirona, Allan Blair Cancer Centre, Regina; Dr CKO Williams, Allan Blair Cancer Centre, Regina; Dr A Maksymiuk, Saskatoon Cancer Centre, Saskatoon; Dr A Wong, Tom Baker Cancer Centre, Calgary; Dr A Fields, Cross Cancer Institute, Edmonton; Dr H Rayner, BCCA – Vancouver Island Cancer Centre, Victoria; Dr B Weinerman, BCCA – Vancouver Island Cancer Centre, Victoria; Dr JE Krook, SMDC Cancer Centre, Duluth; Dr RM Rowland, NCCTG Operations Office, Rochester; Dr MM Veeder, NCCTG Operations Office, Rochester.

*AG9401* Professor AS Coates, Royal Prince Alfred Hospital, Sydney; Professor MPN Findlay, Royal Prince Alfred Hospital, Sydney; A/Professor DR Bell, Royal North Shore Hospital, Sydney; Dr H Gurney, Westmead Hospital, Sydney; Dr B Brigham, Prince of Wales Hospital, Sydney; Dr C Lewis, Prince of Wales Hospital Sydney; Professor ML Friedlander, Prince of Wales, Sydney; Dr D Dalley, St Vincent's Hospital Sydney; A/Professor J Grygiel, St Vincent's Hospital, Sydney; Dr S Della-Fiorentina, Liverpool Hospital, Sydney; Dr E Moylan, Liverpool Hospital, Sydney; Dr A Bonaventura, Newcastle Mater Misercordiae Hospital, Newcastle; Professor SP Ackland, Newcastle Mater Misercordiae Hospital, Newcastle; Dr JF Stewart, Newcastle Mater Misercordiae Hospital, Newcastle; A/Professor E Abdi, Bendigo Hospital, Bendigo; Professor JR Zalcberg, Austin-Repatriation Medical Centre; Dr P Mitchell, Austin-Repatriation Medical Centre; Dr R McIntosh, Townsville Base Hospital, Townsville; Dr J Norman, Queen Elizabeth II Hospital, Adelaide; Dr D Kotasek, Queen Elizabeth II Hospital, Adelaide; Professor R Lowenthal, Royal Hobart Hospital, Hobart; Dr M Jeffrey Christchurch Hospital, Christchurch; Dr M Costello, Dunedin Hospital, Dunedin; Dr D Perez, Dunedin Hospital, Dunedin; Dr S Allan, Palmerston North Hospital, Palmerston North; Dr R Isaacs, Palmerston North Hospital, Palmerston North; Dr G Forgeson, Palmerston North Hospital, Palmerston North.
